# An electrically-controlled programmable microfluidic concentration waveform generator

**DOI:** 10.1186/s13036-018-0126-3

**Published:** 2018-12-14

**Authors:** Joshua Garrison, Zidong Li, Barath Palanisamy, Ling Wang, Erkin Seker

**Affiliations:** 1 Department of Electrical & Computer Engineering, University of California – Davis, Davis, CA 95616 USA; 2 Department of Biomedical Engineering, University of California – Davis, Davis, CA 95616 USA

**Keywords:** Concentration waveform, Pulse width modulation, Microfluidics, Time-varying soluble factors

## Abstract

**Background:**

Biological systems have complicated environmental conditions that vary both spatially and temporally. It becomes necessary to impose time-varying soluble factor concentrations to study such systems, including cellular responses to pharmaceuticals, inflammation with waxing and waning cytokine concentrations, as well as circadian rhythms and their metabolic manifestations. There is therefore a need for platforms that can achieve time-varying concentrations with arbitrary waveforms.

**Results:**

To address this need, we developed a microfluidic system that can deliver concentration waveforms in a fast and accurate manner by adopting concepts and tools from electrical engineering and fluid mechanics. Specifically, we employed pulse width modulation (PWM), a commonly used method for generating analog signals from digital sources. We implement this technique using three microfluidic components via laser ablation prototyping: low-pass filter (lower frequency signals permitted, high frequency signals blocked), resistor, and mixer. Each microfluidic component was individually studied and iteratively tuned to generate desired concentration waveforms with high accuracy. Using fluorescein as a small-molecule soluble factor surrogate, we demonstrated a series of concentration waveforms, including square, sawtooth, sinusoidal, and triangle waves with frequencies ranging from 100 mHz to 400 mHz.

**Conclusion:**

We reported the fabrication and characterization of microfluidic platform that can generate time-varying concentrations of fluorescein with arbitrary waveforms. We envision that this platform will enable a wide range of biological studies, where time-varying soluble factor concentrations play a critical role. In addition, the technology is expected to assist in the development of biomedical devices that allow precise dosing of pharmaceuticals for enhanced therapeutic efficacy and reduced toxicity.

**Electronic supplementary material:**

The online version of this article (10.1186/s13036-018-0126-3) contains supplementary material, which is available to authorized users.

## Background

Time-varying concentrations of soluble factors play an essential role in proper functioning of living systems. A well-known example of this is insulin. While cells respond to spikes of insulin concentration in blood by increasing cellular uptake of glucose, steady-levels of insulin desensitize cells and reduce glucose uptake [1]. There is also a large interest in studying how dynamic extracellular signals can be transduced into intracellular signals and give rise to emergent properties [2, 3]. Furthermore, an expanding body of research reveals the importance of circadian rhythms on inflammation and metabolism [4, 5]. In order to model these complex dynamic biological processes, there is a need for sensors and actuators that can monitor and deliver time-varying concentrations of soluble factors [6]. Even though both the sensor and actuator components are equally important, the focus here is the latter and progress on the former can be found elsewhere [7–9]. One way to categorize the waveform generators is with respect to their concentration pattern output, namely: digital concentration waveforms (i.e., binary/pulsatile switching, which may be relevant for modeling insulin delivery [10]) and analog concentration waveforms (i.e., continuous manipulation of the amplitude and/or frequency, which may be relevant for cytokine patterns following injury [11]). To generate a digital concentration waveform, the general approach is based on switching between two or more liquid inlets, analogous to a multiplexer in electronics, such as peristaltic pumps [12], acoustically vibrating bubbles [13], and magnetic stir rods [14], as well as passive mixers including serpentine channels and herringbone structures [15, 16]. A shortcoming to these approaches is their slow and unpredictable temporal response. Moreover, these designs greatly limit the application that it is only able to create time-varying concentration pulses rather than dynamically changing concentration waveforms. In order to deliver smoothly-varying concentration waveforms, different methods have been devised, including flow control via gas-pressure gated valve and pulse width modulation. However, the improved control of concentration waveforms has come with the expense of system complexity such as gas pipeline, fluid channel network array, many inlets/outlets structure and waste outlets to avoid flow interruptions [17]. We envision that a scalable platform that can deliver concentration waveforms that can be customized by the user would provide an avenue to study complex biological processes. To that end, we developed a microfluidic system that can modulate the concentration waveforms in a fast and accurate manner via pulse width modulation (PWM) that was controlled by electrical signals.

## Results and discussion

**Fig. 1:**
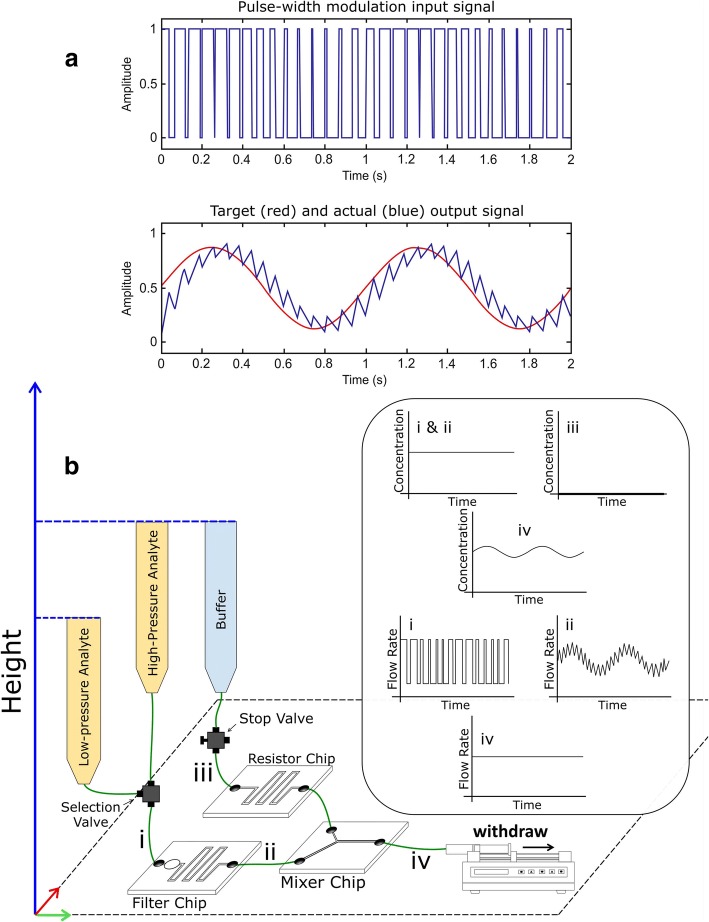
**a**) Conceptual description of pulse width modulation (PWM) technique: A *target signal* (e.g., red sinusoidal wave in bottom plot) is converted to a *PWM signal* (top plot) via a mathematical operation. Low-pass filtering the PWM signal yields the *actual signal* (blue ragged sinusoidal wave in bottom plot) that approximates the red sinusoidal target signal. **b**) Microfluidic integration of the PWM technique: A PWM signal (coding for a sinusoidal target signal as an example) electronically actuates the flow *selection valve* that switches between the *high-pressure* and *low-pressure* analyte reservoirs at the same concentration yet at different hydrostatic pressures due to their height differential. The PWM signal (i.e., pulse train of fast and slow flow rates at node “i”) is converted to the target flow rate signal (at node “ii”) via the *filter chip*. The analyte with the time-varying flow rate combines with the buffer solution at the *mixer chip,* effectively converting the time-varying flow rate signal to a time-varying concentration signal (note “iv”). In order to have constant flow rate at node “d”, a syringe pump withdraws the liquid at a constant flow rate from the *filter chip* and the *resistor chip.* The time-varying concentration and flow rate profiles at four different nodes (i: entering filter chip; ii: leaving filter chip and entering mixer chip; iii: entering resistor chip; iv: leaving mixer chip) are shown inside the rounded rectangular box

It is worth describing PWM operation before dwelling into its implementation. PWM is a prevalent technique in electrical engineering typically used for controlling power transmission to electrical components in applications such as dimming of light-emitting-diode (LED) lamps [18] and servo motors for robotic manipulators [19]. The basic operation principle is that instead of varying the amplitude of a signal with respect to time to generate an arbitrary waveform (e.g., sinusoidal wave); for PWM, pulses with either a high or low fixed amplitude but of varying durations (hence pulse width modulation) is used to generate the desired signal. A common example is a heating element (e.g., electric stove), where the heat delivery is adjusted by varying the on/off duration (“on” duration typically referred to as the *duty cycle*). In its hydraulic analogy, the PWM can be imagined as varying the duration of a reagent delivery (with fixed flow rate) into a liquid stream with a steady-flow rate. The modulation of the duty cycle in turn varies the concentration in the stream (similar to a titrator). One would quickly notice that for a practical application, only the slow-acting (average signal) is desired, which underlines the need for a low-pass filter that removes the undesirable spikes from the individual pulses. Mathematically, this is equivalent to integrating the digital pulse train of varying pulse widths to obtain an analog signal of time-varying amplitude, as shown in Fig. 1a. Specifically, a PWM pulse train (top plot) is obtained through a mathematical operation (see MATLAB code in Supporting Information) that corresponds to the *target* signal (red sinusoidal wave in bottom plot). The PWM input signal is then swept through a low-pass filter to obtain the *actual* output signal (blue ragged sinusoidal wave in bottom plot) that approximates the target signal. Here, we will discuss the implementation of this technique into a fluidic system and its characterization.

### Microfluidic system integration

The electronic-hydraulic analogy allows for applying this electrical concept to fluidics (Additional file 1: Figure S1), as discussed elsewhere [20, 21]. Briefly, a fluidic resistor is a microfluidic channel with specific dimensions to restrict fluid flow while a fluidic capacitor is a chamber with a flexible membrane that can store liquid scaled with respect to the liquid pressure [22]. The proposed microfluidics concentration waveform generator system utilizes three different microfluidic chips (Fig. 1b): (i) *filter chip,* (ii) *resistor chip and* (iii) *mixer chip.* The *filter chip* consists of an elastic membrane-capped cavity as the capacitor and a serpentine channel as the resistor. The *resistor chip* contains a serpentine channel design and the *mixer chip* contains a simple Y-shape channel design. The digitization of the desired output signal (generation of the pulse train with specific pulse widths, that is, pulse width modulation) is performed using a MATLAB algorithm (shown in Supporting Information). The pulse train is then applied through a set of high-current switches (Maxim Integrated) to control the flow selection valve. As shown in Fig. 1b, solutions containing molecules of interest with the same concentration are kept in two reservoirs that are labeled as the *high-pressure analyte* reservoir and *low-pressure analyte* reservoir (shown in yellow). These two reservoirs are connected through a selection valve to the inlet of the *filter chip* and placed at different heights in order to generate different hydrostatic pressures. When the flow selection valve is controlled to switch between these two solutions, even though the concentrations of these two solutions are the same, the output instantaneous flow rates are different, which leads to different volumes of the solution flowing into the *filter chip* per unit time. The *filter chip then acts as* a *low-pass filter* to attenuate the high frequency components originating from the PWM signal and produce an analog output signal of flow rate proportional to the time average of each pulse.

A reservoir filled with the buffer (shown in blue) is connected to the inlet of the *resistor chip* through an adjustable stop valve that allows the flexibility to manually switch out the solution. The buffer from the *resistor chip* is used to generate different waveforms by controllably diluting the solution from the *filter chip* and the final mixing of the solution with the desired concentration waveform is achieved on the *mixer chip*. A syringe pump is connected to the outlet of the *mixer chip* and withdrawing the liquid at a constant rate. Thus, the final mixed solution in the *mixer chip* is at a steady flow rate with the pre-programmed (via PWM pulse train) concentration waveform. Maintaining a constant flow rate while varying the concentration of the solution is not trivial, yet extremely important since in biological experiments the flow rate can influence adherent cell response via hydrodynamic shear forces [23]. Put another way, as solutions from *filter chip* and *resistor chip* enter the *mixer chip* together*,* the sum of the individual flow rate out of *filter chip* and *resistor chip* equals to the final flow rate in the *mixer chip,* which is a constant number programmed by the syringe pump. In order to generate an even flow split between the analyte and buffer on the *mixer chip* at the low-concentration state (flow selection valve not controlled), the microfluidic channel resistance between the *resistor chip* and *filter chip* as well as the hydrostatic pressure of the liquid between *main analyte* reservoir and *buffer* reservoir are the same. Therefore, the *main analyte* reservoir and *buffer* reservoir were placed at same height. Taking sinusoidal concentration waveform as an example, desired concentration waveform, the concentration and flow rate profiles with respect to time at four different nodes (i: entering *filter* chip; ii: leaving *filter* chip and entering *mixer* chip; iii: entering *resistor* chip; iv: leaving *mixer* chip) are shown inside the box in Fig. 1b. These three microfluidics chips in the system can be individually optimized, allowing for improving the overall system performance. The experimental setup of the entire system can be seen in Additional file 1: Figure S2 in supporting information (SI).

In order to facilitate the characterization of the system, we used fluorescein (a small-molecule drug surrogate) for the analyte and deionized (DI) water for the buffer throughout the experiments to allow monitoring the concentration variations with high spatial and temporal resolution. An inverted fluorescence microscope was used to record a short time-lapse video or capture a series of images. The images or the video frames were then uploaded to ImageJ (NIH freeware for image analysis) and the corresponding fluorescence intensity was converted into a gray-scale value and was plotted via MATLAB for post-data analysis (script shown in SI).

### Filter Chip characterization

The *filter chip* is used for producing an analog output waveform by removing high-frequency components of the PWM waveform resulting from the bimodal flow selection valve. In designing the *filter chip*, we utilized a first-order resistor-capacitor (RC) low-pass filter (LPF), which consisted of the microfluidic channel as the resistor and a silicone membrane-capped cavity as the capacitor, as reported elsewhere [22, 24]. We used an elastomer, polydimethylsiloxane (PDMS), as the membrane material and a thin PDMS membrane was bonded on a glass slide covering a cavity hole to form a capacitor. The resistance was controlled by changing the channel dimensions, while the capacitance was adjusted by varying the diameter of the membrane. The fabricated *filter chip* can be seen in Additional file 1: Figure S4 and the cross-sectional schematic can be seen in Additional file 1: Figure S3b in the supporting information. In order to minimize the influence of parasitic capacitances (due to mechanically-compliant components) on the performance of the *filter chip*, rigid glass was used as the substrate and rigid polyetheretherketone (PEEK) tubing was used for connections.

**Fig. 2:**
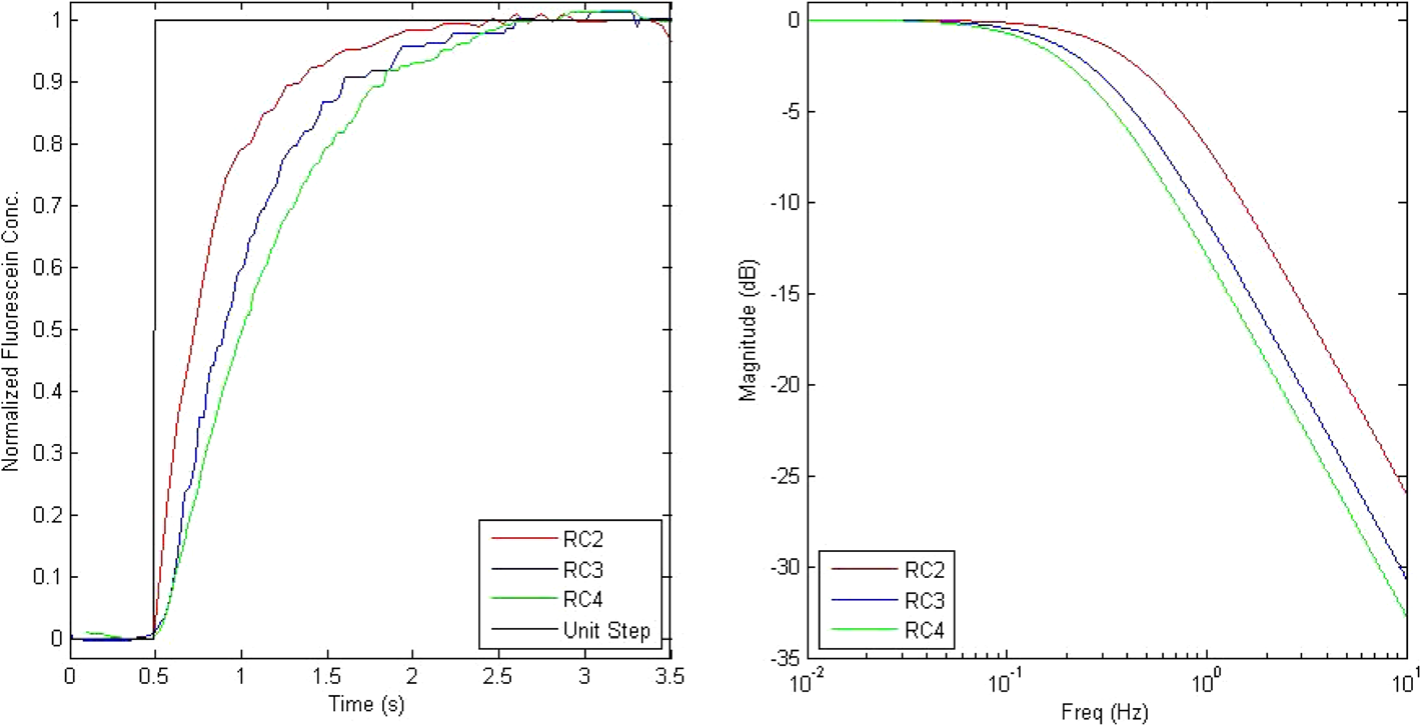
Time response and frequency response of the low-pass filters RC2, RC3, and RC4

Three *filter chips* with the same resistance but different capacitances were fabricated and characterized. The capacitance was varied by changing the cavity diameter (hole diameter covered by the flexible membrane). The diameter of the hole that forms the capacitor was 2 mm, 3 mm, and 4 mm respectively and denoted by RC2, RC3, and RC4. The filter performance in time and frequency domains was characterized by monitoring its response to a step function (extracted from a 100 mHz square waveform), as shown in Fig. 2.

Time constant and cut-off frequency are two important parameters to evaluate the filter performance. Time constant can be determined from the time response plot that equals to the time when the solution reach 63.2% of the target concentration. From the value of time constant *τ*, cut-off frequency *f*_*c*_ can be calculated from Eq. 1.

1 $$ {f}_c=\frac{1}{2\pi \tau} $$

Due to the imperfections in microfabrication process, the theoretical resistance and capacitance from the microfluidic components slightly deviate from the measured parameters. The actual resistance of the chip can be measured by gravity-induced flow and the actual capacitance then can be calculated from Eq. 2, where *τ* is the time constant, *R* is the resistance, and *C* is the capacitance.

2 $$ \tau =R\bullet C $$

As shown in Table 1, the increase in hole diameter led to an increase in capacitance and decrease in cut-off frequency, consistent with expected RC circuit characteristics, illustrating the strength in using electrical component analogies to engineer a fluidic system.

**Table 1:** The experimentally-measured parameters of the three low-pass filter chips

Filter Chip	Designed Diameter D_0_ (mm)	Experimental Time Constant τ (sec)	Experimental Cut-off Frequencies f_c_ (Hz)	Experimental Capacitance C (m^3^/Pa)
RC2	2	0.316	0.504	1.449 × 10^−14^
RC3	3	0.541	0.294	1.693 × 10^−14^
RC4	4	0.687	0.232	2.150 × 10^−14^

### Mixer Chip characterization

The fluorescein solutions from *high-pressure analyte* reservoir and *low-pressure analyte* reservoir were controlled by the selection valve to flow into the *filter chip* and mix with the DI water from the *resistor chip*. The final mixed solution with the desired fluorescein concentration waveform was eventually achieved on the *mixer chip*. The mixing efficiency of the *mixer chip* determines how fast (i.e., within less channel length) the desired concentration waveform can be obtained.

**Fig. 3:**
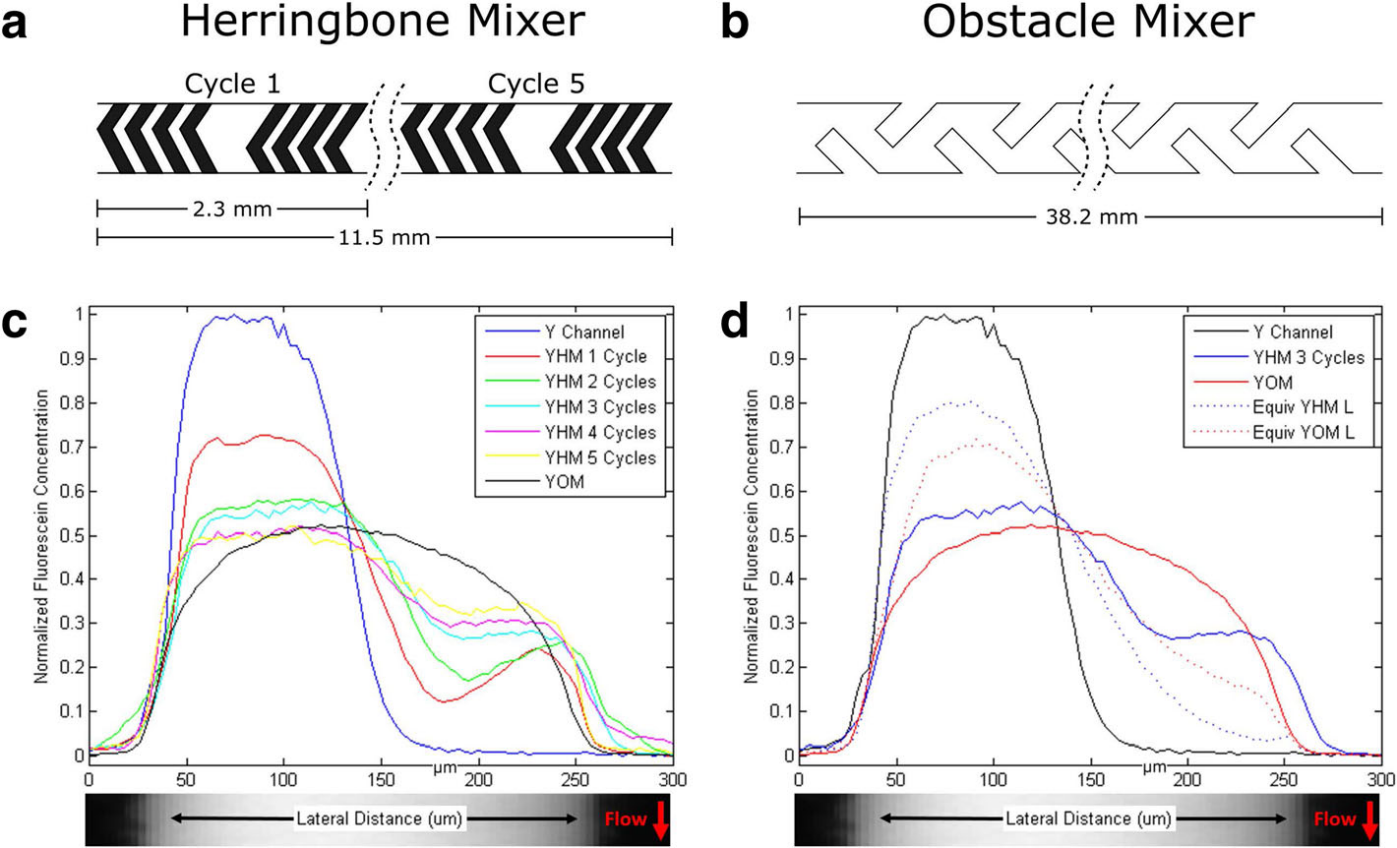
Schematic illustration and lengths of **a**) the *herringbone mixer* (YHM) and **b**) *obstacle mixer* (YOM). **c**) Distribution profiles of fluorescein concentration along the channel width measured at the Y-channel junction of the *mixer chip* (used as the peak fluorescence intensity for normalization), after different numbers of pattern repetitions (cycle) for the *herringbone mixer* (YHM), and after *obstacle mixer* (YOM). **d**) Distribution profiles of fluorescein concentration along the channel width for contrasting the influence of mixers (both YHM 3 Cycles and YOM) with the channels of equivalent length (6.9 mm and 38.3 mm respectively) without any mixer patterns

A fundamental challenge of mixing in microfluidics is the laminar flow conditions, which limits the mixing to solely diffusive transport [25]. In order to increase the mixing efficiency, we employed micro-texturing of the channels following the *Y-junction* (abbreviated as Y Channel). Two different designs were evaluated: (i) three-dimensional herringbone-based mixer (*herringbone mixer*, abbreviated as YHM) and (ii) obstacles patterned on the channel with negative 45 degrees against each other (*obstacle mixer*, abbreviated as YOM), as shown in Fig. 3 and Additional file 1: Figure S5. The general idea behind micro-texturing is to introduce chaotic flow that facilitates convective mixing of the solutions. In order to evaluate the mixing performance of these two designs, 0.05 mM fluorescein solution was used as the target analyte to mix with DI water. This strategy resulted in clear evaluation of the mixing performance under fluorescence microscope as the fluorescein solution appeared bright and the DI water appeared dark. The fluorescence intensity of the liquid inside the channel can be directly correlated to the actual concentration of fluorescein. The mixing efficiency was assessed from the brightness distribution across the channel width via the captured image at downstream of the channel. More specifically, as a semi-quantitative assessment of the mixing efficiency, we performed a full-width at half-maximum (FWHM) analysis for the fluorescein distribution profiles shown in Fig. 3. The results are illustrated in Additional file 1: Figure S6.

We first characterized and evaluated the *herringbone mixer* to study the mixing efficiency with different numbers of pattern repetitions. Each number of pattern repetitions (also referred to as cycle) of the herringbone structure is 2.3 mm long and five different chips with five unique numbers (one through five) were tested. As it can be seen from the distribution of fluorescein concentration across the channel width (Fig. 3c), the Y-channel control (at the Y-channel junction) is highly ineffective at creating a uniform concentration along the channel width, as confirmed by the FWHM analysis (Additional file 1: Figure S6). This is also apparent as the width of high fluorescence intensity region (high concentration of fluorescein) after the junction is roughly the half of the entire channel width (Fig. 3c), indicating that the two solutions were not mixed thoroughly. The inclusion of herringbone mixer patterns improved mixing efficiency due to the circular vortexes that accompany the off-center grooves [16]. Since there was no significant improvement in the mixing efficiency for the herringbone structures for more than three-pattern repetitions (as shown in Additional file 1: Figure S6), the three-pattern repetition architecture was chosen*. Obstacle mixer,* albeit a much longer channel (38.3 mm), also enabled robust mixing (Additional file 1: Figure S6). For this design, the negative obstacle angles create chaotic flow by manipulating flow towards the center of the channel and lead to effective mixing [26]. While the o*bstacle mixer* exhibited more uniform mixing than the *herringbone mixer* along the width of the channel, this was at the expense of a significantly longer time (~ 5 times longer channel), which may be impractical for chip lay-out. In contrast, plain channels (without any mixer patterns) with equivalent lengths to the three-pattern herringbone mixer and the obstacle mixer (shown respectively as *Equiv YHM L* and *Equiv YOM L* in Fig. 3d and Additional file 1: Figure S6) displayed poor mixing due to the purely diffusive mixing mechanism available. As the outcome of the mixer chip characterization, three-pattern cycle version of the *herringbone mixer* was chosen as the final mixer chip component.

An important characteristic of mixers is that they can also be characterized as low-pass filters that attenuate high-frequency waveforms and do not affect low-frequency waveforms. While this further smooths out the output signal (waveforms at nodes “ii” and “iv” in Fig. 1b), it may lead to smearing of the waveform. This becomes more significant for longer mixing times (e.g., longer mixer channels, such as the YOM), since dispersion (due to diffusion along the channel length) further broadens the concentration waveforms and reduces the peak concentrations [27]. The detailed discussion and its mathematical treatment can be found in the supporting information. The time response and frequency response of *herringbone mixer* and *obstacle mixer* is shown in Additional file 1: Figure S7.

### Concentration waveform generation

**Fig. 4:**
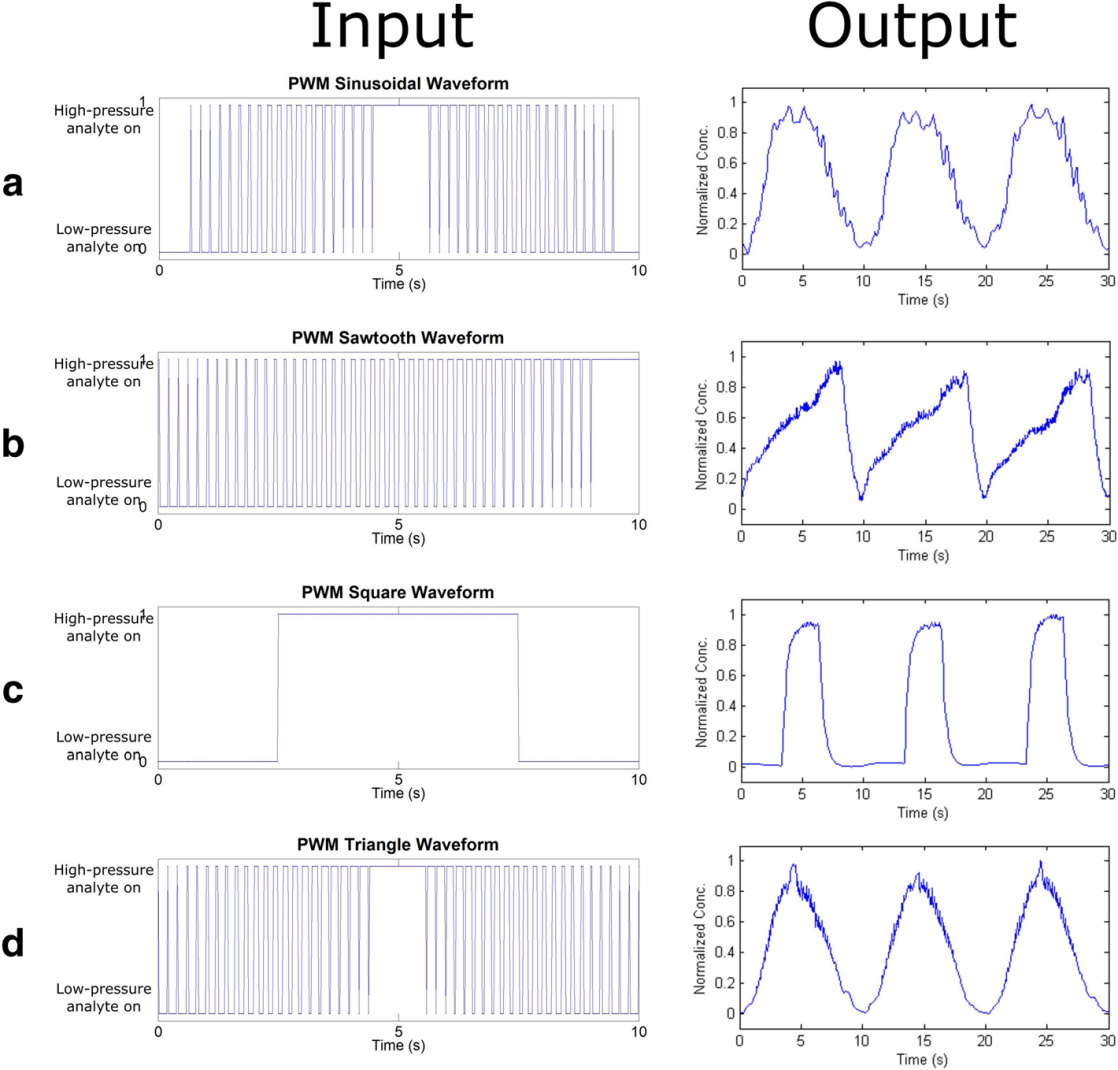
Different concentration waveforms of 100 mHz are generated from the microfluidic system: **a**) sinusoidal, **b**) triangle, **c**) sawtooth, and **d**) square waves, as shown in right panel. The corresponding PWM signal used for switching between the *high-pressure analyte* and *low-pressure analyte* reservoirs are shown in the left column. Note that only one period of the PWM signal is shown for clarity

Following careful characterization and optimization of the individual components necessary for generating concentration waveforms, we assembled the microfluidic system as shown in Fig. 1b. With the RC2 *filter chip* and same length *resistor chip* as well as three-pattern repetitions of *herringbone mixer*, a variety of waveforms can be generated. As shown in the right column of Fig. 4, sinusoidal, triangle, sawtooth, and square concentration waveforms with 100 mHz were generated by the system. The corresponding PWM signals that control the switching between the reservoirs of *high-pressure analyte* and *low-pressure analyte* are shown in the left column. Since the frequency of the applied electrical signals can also help change the shape of the generated waveforms, it is possible to tune the waveforms by simply controlling the frequency without having to change the physical system components. As the frequency increases, the sawtooth waveform begins to morph into a triangle wave (Additional file 1: Figure S8) while the square waveform shows sharper and more frequent peaks (Additional file 1: Figure S9). With a combination of these basic concentration waveform primitives, other more complicated concentration waveforms can be generated, highlighting the versatility of the platform.

In the current microfluidic system, the cut-off frequencies of the *filter chip* are between 200 mHz and 500 mHz whereas the *mixer chip* are between 15 mHz and 70 mHz. The *mixer chip* limits the speed of the final concentration waveform as it has a significantly lower cut-off frequency than any of the filters. However, the channel length correlates with the cut-off frequency, thus a shorter mixer chip can be used for faster response. Depending on the application and the desired waveform’s characteristic, different filters and mixers could be tuned easily to obtain very specific concentration waveforms.

## Conclusions

We presented a microfluidic concentration waveform generator by adopting techniques and tools from electrical engineering and fluid mechanics. Specifically, we employed pulse width modulation (PWM) technique enabled by an electrically-controlled flow-selection valve to create flow-rate pulses of a high concentration analyte that were smoothed out by a fluidic first-order low-pass filter before titrating it into a buffer solution at a Y-channel junction, and mixing it via a microtextured channel. Each component was separately characterized before implementation into the system. The system successfully generated fundamental waveforms (e.g., sinusoidal, triangle, sawtooth, square) and a MATLAB algorithm was developed to program more complex arbitrary waveforms.

Having established a system that can create arbitrary concentration waveforms, it is important to conclude by discussing its utility in biology. Biological processes are inherently a product of sophisticated negative and positive feedback loops with different time scales (e.g., phosphorylation versus synthesis of proteins). Per *system identification theory* [28], in order to deconvolve these mechanisms with different time scales, it is necessary to develop tools that can characterize the biological system’s response to soluble factors with different magnitudes and temporal profiles. An emerging area of relevance is the cross-talk between inflammation and metabolism, where cytokines influence metabolic processes (e.g., tumor necrosis factor-alpha and PPAR interaction [29]), which may lead to paradoxical effects like hypermetabolism in cancer and obesity, both of which has an inflammatory component. It is well-documented that cytokines and their temporal response play a significant role in physiological time course following injury and in a large set of diseases [30, 31]. From a more applied perspective, other examples of this phenomenon are widespread in biology such as the tolerance effect exhibited by various drug administrations in which down-regulation of receptor expression can blunt the effect of a drug if the initial dose is given at too high level, or at too rapid of an interval between doses [32, 33]. For a such case, drug dosing at the correct waveform may improve efficacy. Concentration waveforms can also be tuned into a more repeatable pattern to study the circadian rhythms and their influences on inflammation and metabolism in many diseases including atherosclerosis and obesity [4, 5]. Progress in such studies can be translated into pharmacological and/or nutritional interventions with tremendous therapeutic potential. Overall, we expect that the engineered platform will enable a rich set of studies ranging from fundamental biology to translational medicine.

## Methods

### Flow selection valve and pulse width modulation (PWM) signal generation

The flow selection valve is the essential component in the waveform generator system and it controls the flow-rate alternation between the liquid in *high-pressure analyte* reservoir (higher hydrostatic pressure) and *low-pressure analyte* reservoir (lower hydrostatic pressure) to flow into the *filter chip*. It is electrically-controlled, where in order to toggle between the *high-pressure analyte* reservoir and *low-pressure analyte* reservoir, 12 V was applied on only one side and then switched to the other. A pair of high-current switches was used to convert logic signals (PWM pulse train) into 12 V lines to change the state of the valves. The PWM signals for the waveforms of interest were generated by a custom MATLAB algorithm (shown in Supporting Information). The PWM signal was imported into the Analog Discovery’s waveform generator and directly used to control the flow selection valve through switches. This script (see supporting information) can generate sinusoidal, square, and sawtooth waveforms but can easily be adapted for any waveform.

### Channel resistance measurement by gravity induced flow

The microfluidics channel resistance was measured by gravity-induced flow. Simply, the solution was filled in a reservoir that placed at a fixed height above the chip and the resistance of the chip can be calculated from the flow rate. The hydraulic resistance (*R*_*H*_) through a channel with volumetric flow rate *Q* results in a pressure drop through the following equation:

3 $$ \varDelta P\kern0.5em =\kern0.5em {R}_{\mathrm{H}}Q $$

Furthermore, by using gravity (*g*) induced flow with a reservoir of solution with density (*p*) at a height (h) above the inlet of a microfluidic chip, a pressure drop can be calculated through the following equation:

4 $$ \varDelta P\kern0.5em =\kern0.5em \rho \mathit{\mathsf{g}}\mathrm{h} $$

Combining Eqs. 3 and 4, it allows for a simple calculation to find the resistance of a microfluidic channel and tubing.

### Microfluidic Chip fabrication

**Fig. 5:**
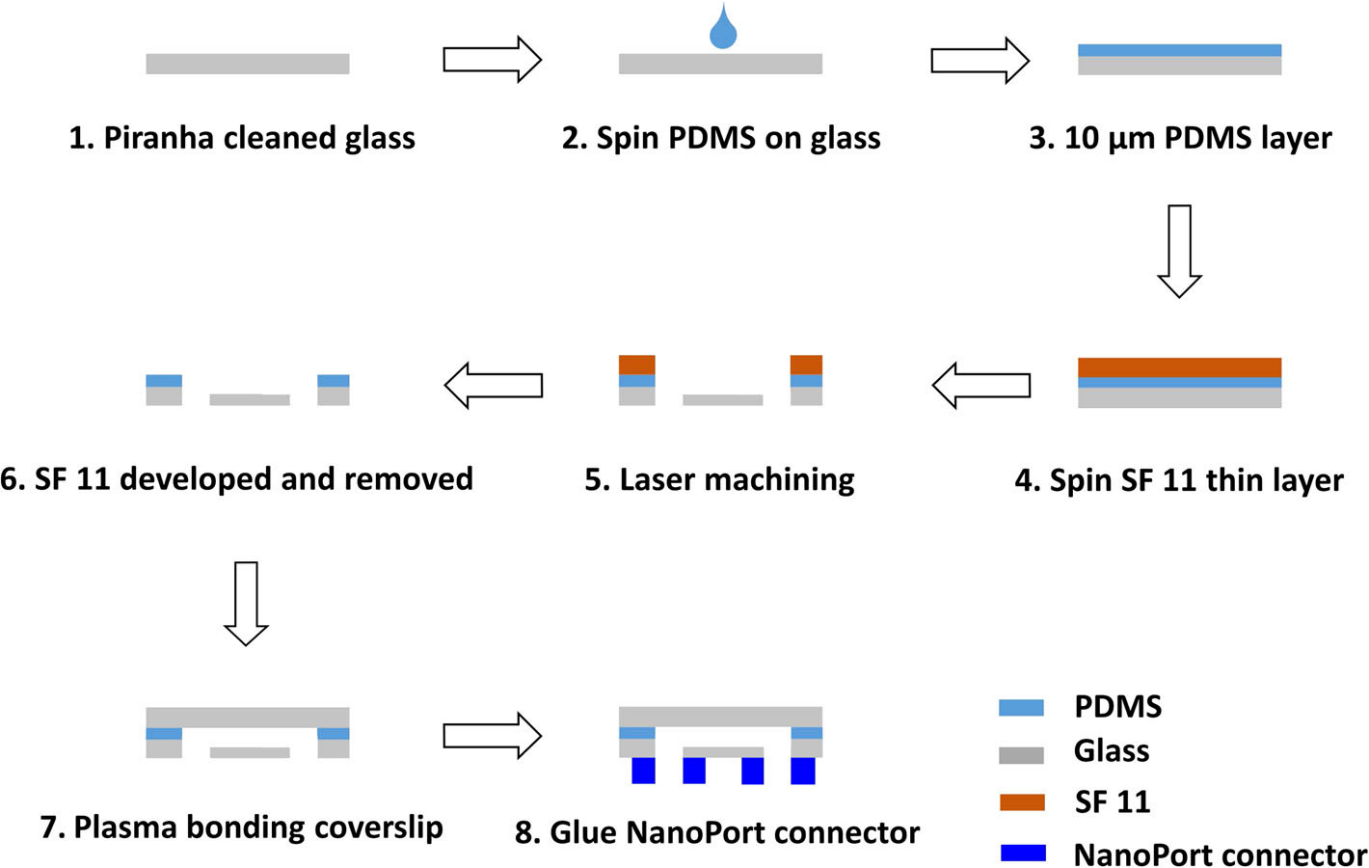
The fabrication process steps for the microfluidic device

In this microfluidic system, three chips were fabricated and tested including the *filter chip*, *resistor chip*, and the *mixer chip* (with herringbone structure and obstacle structure). The cross-sectional schematics of the three chips are shown in Additional file 1: Figure S3. The fabrication of all microfluidic chips in this system relied on a simple and robust 355 nm UV laser ablation instead of traditional photolithography [34]. The laser-patterned device was then bonded to another glass substrate (0.15 mm-thick coverslip or 1 mm-thick glass slide) through a 10 μm-thick PDMS intermediate adhesive layer. As shown in the fabrication process flow in Fig. 5, PDMS pre-mixer solution (1:10 *w*/w curing agent to base) was coated on a glass slide to produce a uniform 10 μm-thick PDMS layer. A thin SF-11 protective layer was coated on the PDMS to prevent debris that were generated during the laser-cutting process. After laser cutting, the SF-11 coated device was immersed in developer solution to remove SF-11 layer. Then the device was bonded to another laser-machined glass substrate under oxygen plasma (0.5025 Torr, 20 sccm O_2_, 30 W) to achieve the final microfluidic device.

The specific channel depth and width vary for each chip. In the *mixer* chip, the channel depth and width are 200 μm. The *filter* and *resistor* chips have a smaller channel depth and width of 100 μm, as a much higher resistance is needed for the two chips to produce fast waveforms. The channel depths and widths were measured by a profilometer and confirmed via a differential interference contrast (DIC) optical microscope. Subsequently, NanoPort connectors (Western Analytical Products) were glued onto the inlets and outlets of the microfluidic chips for tubing connection.

### Data analysis for time and frequency responses

The time and frequency responses were analyzed for the *filter chip* and *resistor chip*. For determining the *time-constant* and *cut-off frequency*, MATLAB algorithms were used. Briefly, MATLAB was used to separate a full waveform into equivalent sections and average them to accurately find step and frequency responses. Subsequent analysis was completed on the short output waveforms obtained from this script (see supporting information) instead of the full waveforms. This script finds the first period of a waveform and uses it as a template in cross-correlation with the entire waveform. The highest values obtained from cross-correlation are the more-closely-matched sections of the waveform to the template. Each section is then averaged together to find the step and frequency response. Detailed information about the MATLAB code can be found in the supporting information.

### Overall system evaluation and characterization

Fluorescein solution and deionized water were used in the system to demonstrate the generation of programmed concentration waveforms. As the fluorescein solution appears bright and the deionized water appears dark under an inverted fluorescence microscope (Zeiss Observer D1), the intensity of the liquid inside the channel can be directly correlated to the actual concentration of fluorescein via a calibration curve. The fluorescence microscope was used to record a short time-lapse video or capture a series of images. Each video sample or the image sample was then uploaded to ImageJ and the corresponding brightness was extracted into a gray value and then was plotted through MATLAB for post-data analysis, including the full-width at half-maximum extraction (Additional file 1: Figure S6).

## Additional file

Additional file 1: **Figure S1.** Electronic-hydraulic analogy of microfluidic resistor and microfluidic capacitor. **Figure S2.** The experimental setup of the microfluidics system. **Figure S3.** The cross-sectional schematics of the *resistor chip*, *filter chip* and *mixer chip*. **Figure S4.** The *filter chip* with PDMS deformable membrane as a capacitor. **Figure S5.** The schematic depicts obstacle pattern and herringbone micro-texturing inside the microfluidic channel. **Figure S6.** Full-Width at Half-Maximum analysis of mixing efficiency. **Figure S7.** Time and frequency response of *herringbone mixer* and *obstacle mixer*. **Figure S8.** Comparison of 100 mHz, 200 mHz, and 400 mHz sawtooth waveforms from RC2. **Figure S9.** Comparison of 100 mHz and 400 mHz square waveforms from RC2. (PDF 433 kb)

## Abbreviations

DI: Deionized
DIC: Differential interference contrast
LED: Light-emitting-diode
LPF: Low-pass filter
PDMS: Polydimethylsiloxane
PEEK: Polyetheretherketone
PWM: Pulse width modulation
RC: Resistor-capacitor
sccm: standard cubic centimeters per minute

## References

[1] Buren J, Liu HX, Lauritz J, Eriksson JW (2003). High glucose and insulin in combination cause insulin receptor substrate-1 and -2 depletion and protein kinase B desensitisation in primary cultured rat adipocytes: possible implications for insulin resistance in type 2 diabetes. Eur J Endocrinol.

[2] Mettetal JT, Muzzey D, Gomez-Uribe C, van Oudenaarden A (2008). The frequency dependence of osmo-adaptation in Saccharomyces cerevisiae. Science.

[3] Jovic A, Wade SM, Miyawaki A, Neubig RR, Linderman JJ, Takayama S (2011). Hi-fi transmission of periodic signals amid cell-to-cell variability. Mol BioSyst.

[4] Turek FW, Joshu C, Kohsaka A, Lin E, Ivanova G, McDearmon E (2005). Obesity and metabolic syndrome in circadian clock mutant mice. Science.

[5] Lange T, Dimitrov S, Born J (2010). Effects of sleep and circadian rhythm on the human immune system. Ann N Y Acad Sci.

[6] Li Z, Polat O, Seker E (2018). Voltage-gated closed-loop control of small-molecule release from alumina-coated Nanoporous gold thin film electrodes. Adv Funct Mater.

[7] Ferguson BS, Hoggarth DA, Maliniak D, Ploense K, White RJ, Woodward N (2013). Real-Time, Aptamer-Based Tracking of Circulating Therapeutic Agents in Living Animals. Sci Transl Med.

[8] Plaxco KW, Soh HT (2011). Switch-based biosensors: a new approach towards real-time, in vivo molecular detection. Trends Biotechnol.

[9] Bandak B, Yi L, Roper MG (2018). Microfluidic-enabled quantitative measurements of insulin release dynamics from single islets of Langerhans in response to 5-palmitic acid hydroxy stearic acid. Lab Chip.

[10] Porksen N, Hollingdal M, Juhl C, Butler P, Veldhuis JD, Schmitz O (2002). Pulsatile insulin secretion: detection, regulation, and role in diabetes. Diabetes.

[11] Helmy A, Carpenter KLH, Menon DK, Pickard JD, Hutchinson PJA (2011). The cytokine response to human traumatic brain injury: temporal profiles and evidence for cerebral parenchymal production. J Cereb Blood Flow Metab.

[12] Unger MA, Chou HP, Thorsen T, Scherer A, Quake SR (2000). Monolithic microfabricated valves and pumps by multilayer soft lithography. Science.

[13] Ahmed D, Muddana HS, Lu M, French JB, Ozcelik A, Fang Y (2014). Acoustofluidic chemical waveform generator and switch. Anal Chem.

[14] Liang-Hsuan L, Kee Suk R, Chang L (2002). A magnetic microstirrer and array for microfluidic mixing. J Microelectromech Syst.

[15] Aubin J, Fletcher DF, Xuereb C (2005). Design of micromixers using CFD modelling. Chem Eng Sci.

[16] Yang J-T, Huang K-J, Lin Y-C (2005). Geometric effects on fluid mixing in passive grooved micromixers. Lab Chip.

[17] Chen P, Guo Y, Feng X, Yan S, Wang J, Li Y (2017). Microfluidic chemical function generator for probing dynamic cell signaling. Anal Chem.

[18] Chiu H, Lo Y, Chen J, Cheng S, Lin C, Mou S (2010). A high-efficiency dimmable LED driver for low-power lighting applications. IEEE Trans Ind Electron.

[19] Holtz J (1994). Pulsewidth modulation for electronic power conversion. Proc IEEE.

[20] Mosadegh B, Kuo C-H, Tung Y-C, Torisawa Y-S, Bersano-Begey T, Tavana H (2010). Integrated elastomeric components for autonomous regulation of sequential and oscillatory flow switching in microfluidic devices. Nat Phys.

[21] Kim G, Van Dang B, Kim S-J (2018). Stepwise waveform generator for autonomous microfluidic control. Sensors Actuators B Chem.

[22] Leslie D, Easley C, Seker E, Karlinsey J, Utz M, Begley M (2009). Frequency-specific flow control in microfluidic circuits with passive elastomeric features. Nat Phys.

[23] Gallant ND, Michael KE, García AJ (2005). Cell adhesion strengthening: contributions of adhesive area, integrin binding, and focal adhesion assembly. Mol Biol Cell.

[24] Kang YJ, Yang S (2012). Fluidic low pass filter for hydrodynamic flow stabilization in microfluidic environments. Lab Chip.

[25] Whitesides GM (2006). The origins and the future of microfluidics. Nature.

[26] Rasouli M, Abouei Mehrizi A, Lashkaripour A (2015). Numerical study on low Reynolds mixing oft-shaped micro-mixers with obstacles. Transp Phenom Nano Micro Scales.

[27] Beard DA (2001). Taylor dispersion of a solute in a microfluidic channel. J Appl Phys.

[28] Kitano H (2002). Computational systems biology. Nature.

[29] Chinetti G, Fruchart J-C, Staels B (2000). Peroxisome proliferator-activated receptors (PPARs): nuclear receptors at the crossroads between lipid metabolism and inflammation. Inflamm Res.

[30] Tisoncik JR, Korth MJ, Simmons CP, Farrar J, Martin TR, Katze MG (2012). Into the eye of the cytokine storm. Microbiol Mol Biol Rev.

[31] Finnerty CC, Herndon DN, Przkora R, Pereira CT, Oliveira HM, Queiroz DMM (2006). Cytokine expression profile over time in severely burned pediatric patients. Shock.

[32] Portaluppi F, Lemmer B (2007). Chronobiology and chronotherapy of ischemic heart disease. Adv Drug Deliv Rev.

[33] Williams JT, Christie MJ, Manzoni O (2001). Cellular and synaptic adaptations mediating opioid dependence. Physiol Rev.

[34] Li Z, Seker E (2017). Configurable microfluidic platform for investigating therapeutic delivery from biomedical device coatings. Lab Chip.

