# Supplementary material for: An electrically-controlled programmable microfluidic concentration waveform generator
Source: J Biol Eng. 2018 Dec 14;12:31. doi: 10.1186/s13036-018-0126-3 (PMC6295081; doi:10.1186/s13036-018-0126-3)
Supplement: Supplementary file 1 — Figure S1. Electronic-hydraulic analogy of microfluidic resistor and microfluidic capacitor. Figure S2. The experimental setup of the microfluidics system. Figure S3. The cross-sectional schematics of the resistor chip, filter chip and mixer chip. Figure S4. The filter chip with PDMS deformable membrane as a capacitor. Figure S5. The schematic depicts obstacle pattern and herringbone micro-texturing inside the microfluidic channel. Figure S6. Full-Width at Half-Maximum analysis of mixing efficiency. Figure S7. Time and frequency response of herringbone mixer and obstacle mixer. Figure S8. Comparison of 100 mHz, 200 mHz, and 400 mHz sawtooth waveforms from RC2. Figure S9. Comparison of 100 mHz and 400 mHz square waveforms from RC2. (PDF 433 kb) [file 13036_2018_126_MOESM1_ESM.pdf]

Supporting Information for:

An electrically-controlled programmable microfluidic concentration waveform generator

Joshua Garrison[1], Zidong Li[2], Barath Palanisamy[2], Ling Wang[1], Erkin Seker[1,*]

Departments of [1]Electrical & Computer Engineering and [2]Biomedical Engineering

University of California – Davis, Davis, CA 95616

Electronic-hydraulic analogy

The electronic-hydraulic analogy allows for applying the electrical concept to fluidics (**Figure S1**). Briefly, a fluidic resistor is a microfluidic channel with specific dimensions to restrict fluid flow while a fluidic capacitor is a chamber with a flexible membrane that can store liquid scaled with respect to the liquid pressure.

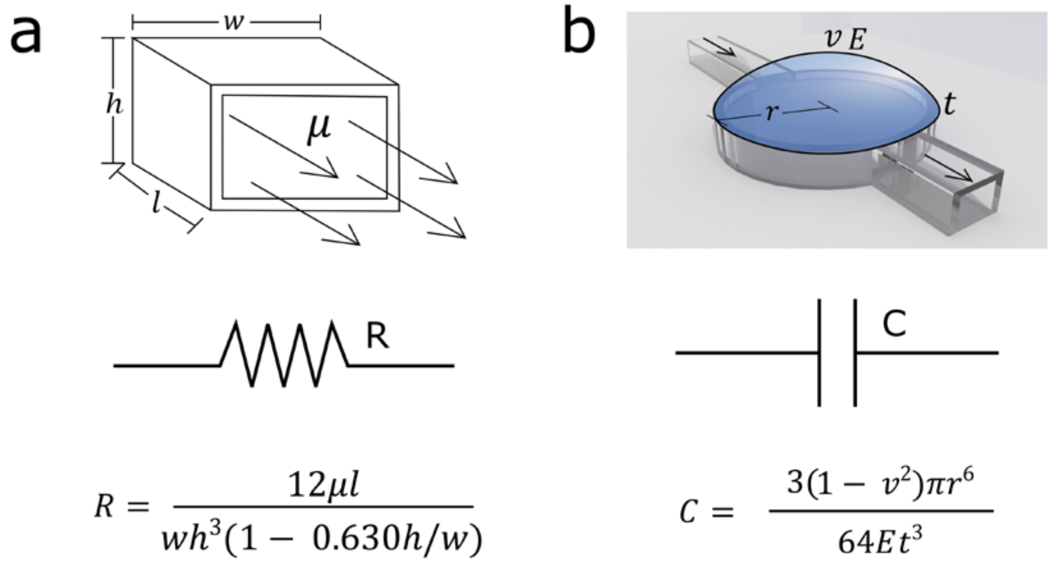

Figure S1. Electronic-hydraulic analogy of **a**) microfluidic resistor and **b**) microfluidic capacitor. In the equations above, *R* is the resistance, *w* is channel width, *h* is channel height, *l* is channel length, μ is the viscosity of the flowing fluid inside the channel; *C* is the capacitance, *r* and *t* are the effective radius and thickness of the fluidic capacitor's membrane (shown in blue), ν and *E* are the Poisson ratio and Young's modulus the membrane material

Microfluidic system integration

The experimental setup of the microfluidic system is shown in **Figure S2**. The *high-pressure analyte* and *low-pressure analyte* reservoirs contain the analyte solution (in this case, fluorescein as a small-molecule surrogate). These two reservoirs are connected through a selection valve to the microfluidic chip to mix with buffer (in this case, deionized (DI) water for simplicity) to generate desired concentration waveforms. The flow rate inside the microfluidic chips was maintained at a constant rate by a syringe pump. In order to toggle between the high-pressure analyte reservoir and low-pressure analyte reservoir, the pulse width modulation (PWM) signal was imported into the Analog Discovery's waveform generator and directly used to control the flow selection valve through the switches. A pair of high-current switches was used to convert logic signals (i.e., PWM pulse train) into 12 V lines to change the state of the selection valve.

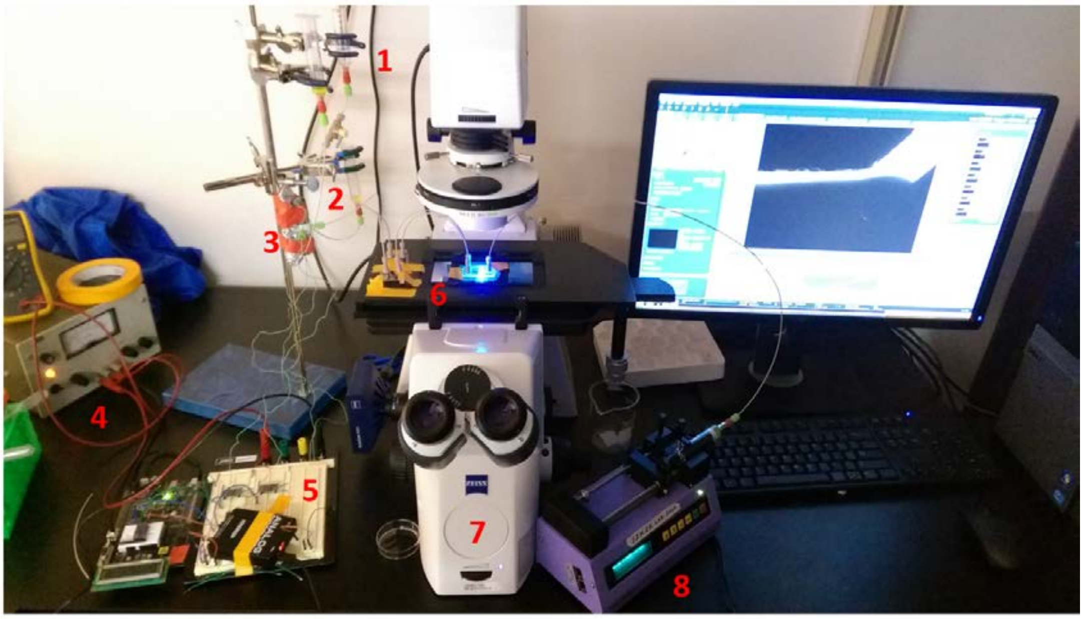

Figure S2. The experimental setup of the microfluidics system, peripheral electronic control circuitry, and fluorescence microscope: 1) High-pressure analyte and buffer reservoirs; 2) Low-pressure analyte reservoir; 3) Flow selection valve; 4) 12 V power supply; 5) PSOC 5LP and Analog Discovery pulse-width modulation circuitry; 6) Microfluidic chip; 7) Fluorescence microscope and camera; 8) Syringe pump

Fabrication of microfluidic chips

The cross-sectional schematics of the three chips, including the *filter chip*, *resistor chip*, and the *mixer chip* (with herringbone structure and obstacle structure) are shown in **Figure S3**. Instead of traditional photolithography, UV laser ablation was used to fabrication all the microfluidic devices. This rapid-prototyping fabrication method is simple and robust with high turn-around.

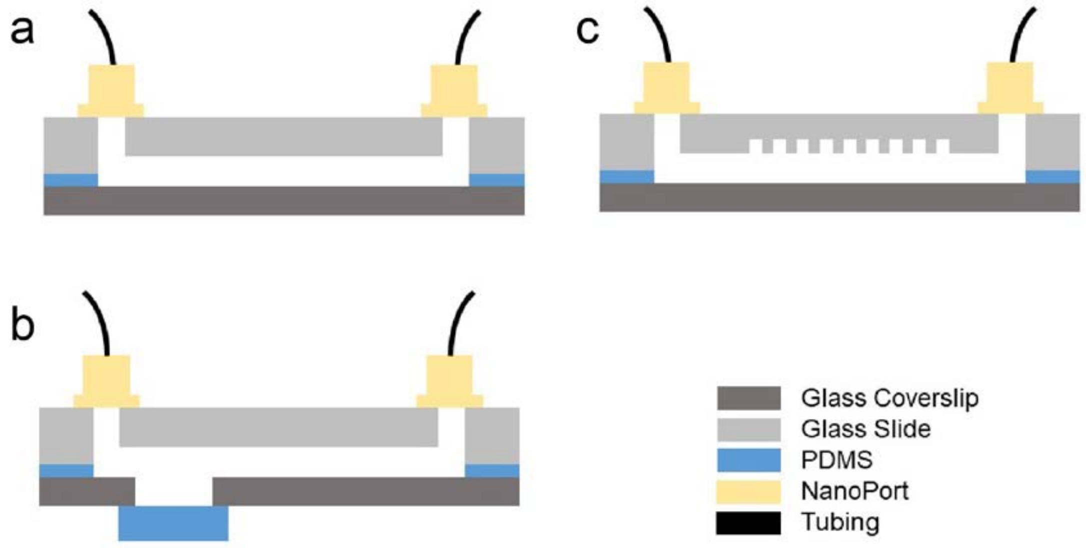

Figure S3. The cross sectional schematics of the **a**) *resistor chip*, **b**) *filter chip* and **c**) *mixer chip*

Filter Chip characterization

The *filter chip* is used to produce a smooth output waveform by removing high-frequency components of the PWM waveform resulting from the flow selection valve. We utilized first-order resistor-capacitor (RC) low-pass filter (LPF), which consisted the microfluidic channel as the resistor and a silicone membrane-capped cavity as the capacitor. The resistance was controlled by changing the channel dimensions, while the capacitance was adjusted by varying the diameter of the membrane. The fabricated *filter chip* can be seen in **Figure S4**. Simply, a thin PDMS membrane was bonded on a glass slide covering a cavity hole to act as a capacitor.

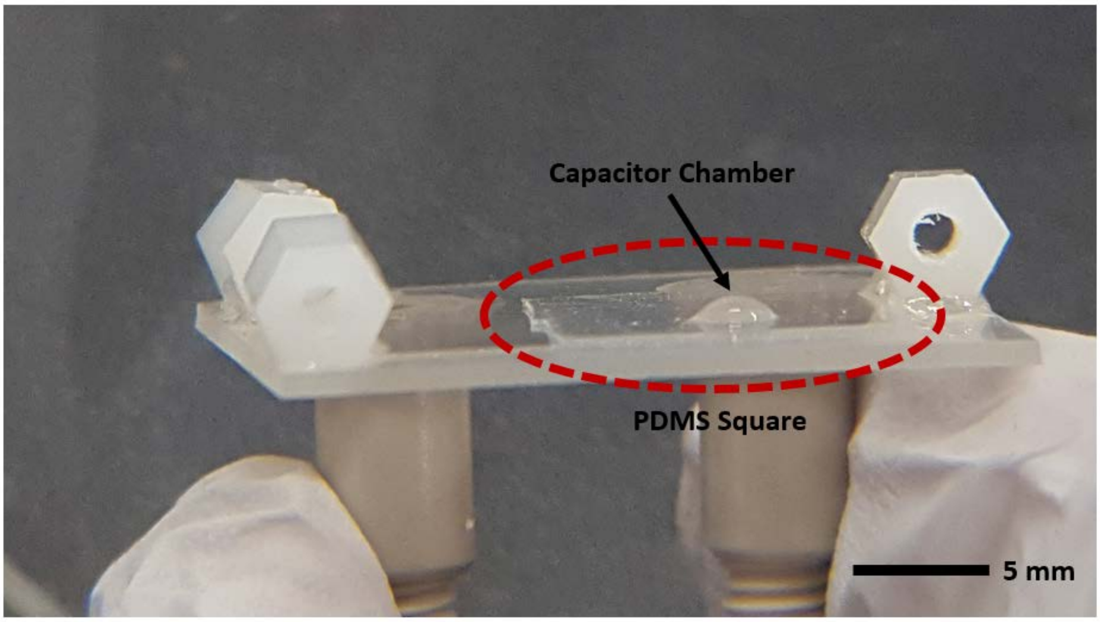

Figure S4. The filter chip with PDMS deformable membrane as a capacitor

Mixer Chip characterization

Two different mixer designs to overcome the challenge of mixing in laminar flow conditions were evaluated (shown in **Figure S5**): (i) three-dimensional herringbone-based mixer (*herringbone mixer*) and (ii) obstacles patterned on the channel with negative 45 degrees against each other (*obstacle mixer*). The general idea behind micro-texturing is to introduce chaotic flow that assists convective mixing of the solutions.

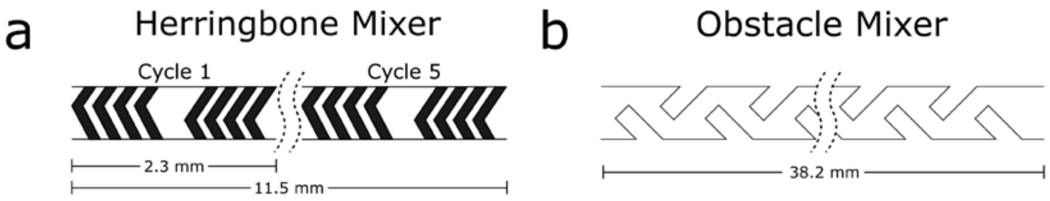

Figure S5. The schematic depicts **a**) herringbone mixer (YHM) and **b**) obstacle mixer (YOM) patterns inside the microfluidic channel

For a semi-quantitative analysis of the mixing efficiency, we employed full-width at half-maximum analysis of the fluorescein distribution patterns along the channel width that are shown in **Figure 3**. As **Figure S6** illustrates, absence of mixer features (Y Channel junction, Equiv YHM L, and Equiv YOM L) lead to poor mixing. The mixing efficiency levels out after three cycles of herringbone patterns (YHM 3 Cycles). While the obstacle mixer (YOM) displays good mixing, the five-times longer channel compared to the three-pattern repetition herringbone mixer may suffer from smearing of concentration patterns long the channel length.

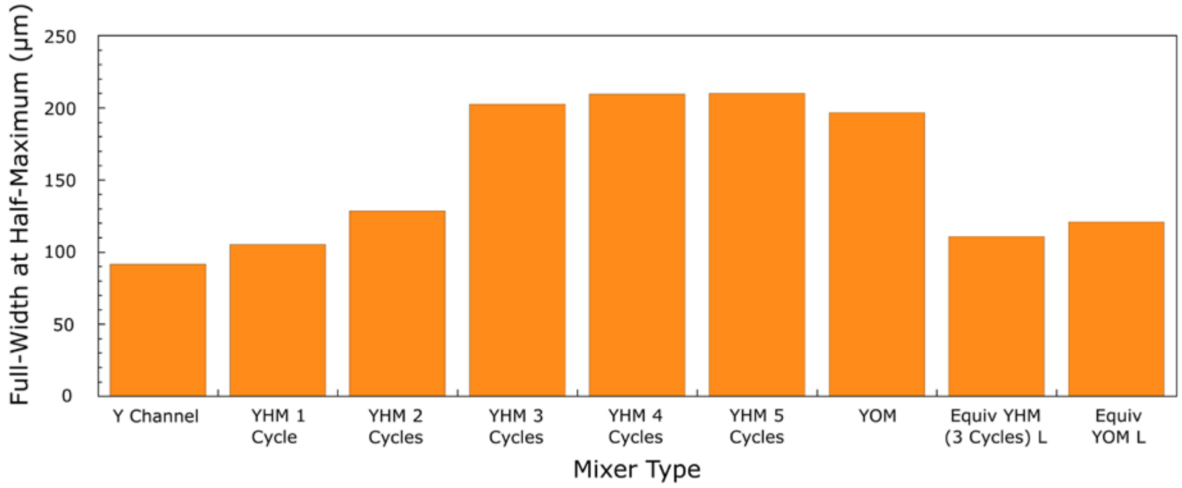

Figure S6. Full-width at half-maximum (FWHM) analysis for the fluorescein distribution profiles shown in Figure 3 in the main manuscript. Plain channels (without any mixer patterns) with equivalent lengths to the three-pattern herringbone mixer and the obstacle mixer were denoted respectively as *Equiv YHM L* and *Equiv YOM L*

An important characteristic of the *mixer chip* is that it can also be characterized as a low-pass filter that attenuate high-frequency waveforms and do not affect low-frequency waveforms.

To a first order approximation, the ratio of filtered output $C(f, L)$ to input $C(f, 0)$ approximately obeys:

$$\left\| \frac{C(f,L)}{C(f,0)} \right\| \approx \exp(\frac{f}{f_c}) \qquad (1)$$

The variables f and f_c are the excitation and cut-off frequencies. For $f > f_c$, input waveforms will be significantly attenuated. The cut-off frequency is approximately:

$$f_c = \frac{1}{2\pi}\left(\frac{V^3}{DL}\right)^{1/2} \qquad (2)$$

where *V*, *D*, and *L* are the flow velocity, diffusion coefficient, and channel length respectively.

Similar to the method for extracting the time response and frequency response from the *filter chip*, a unit step 5 Hz PWM signal with frequencies of 50 mHz was applied on *herringbone mixer* and 10 mHz for the *obstacle mixer* (shown in **Figure S7**). The time constants for the *herringbone mixer* and the *obstacle mixer* are 2.36 sec and 10.50 sec respectively that correlate with the cut-off frequencies of 67.5 mHz and 15.2 mHz with a first-order low-pass filter approximation. *Herringbone mixer* responds approximately five times faster than *obstacle mixer* and is also approximately five times shorter in length. Based upon Equation 2, *herringbone mixer* should only respond $\sqrt{5}$ times faster which means the herringbone structures are significantly more efficient at mixing than the negative angle obstacle structures for a given amount of length. As stated in the main manuscript comparing mixing profiles of equal length of the two mixers, although *obstacle mixer* showed better mixing along the width of the channel, it takes significantly longer to mix thoroughly. Mixing time is an important consideration in determining the efficiency of passive mixers, because a faster mixer eventually can lead to fast concentration waveforms. Therefore, *herringbone mixer* was used as the final mixer chip component.

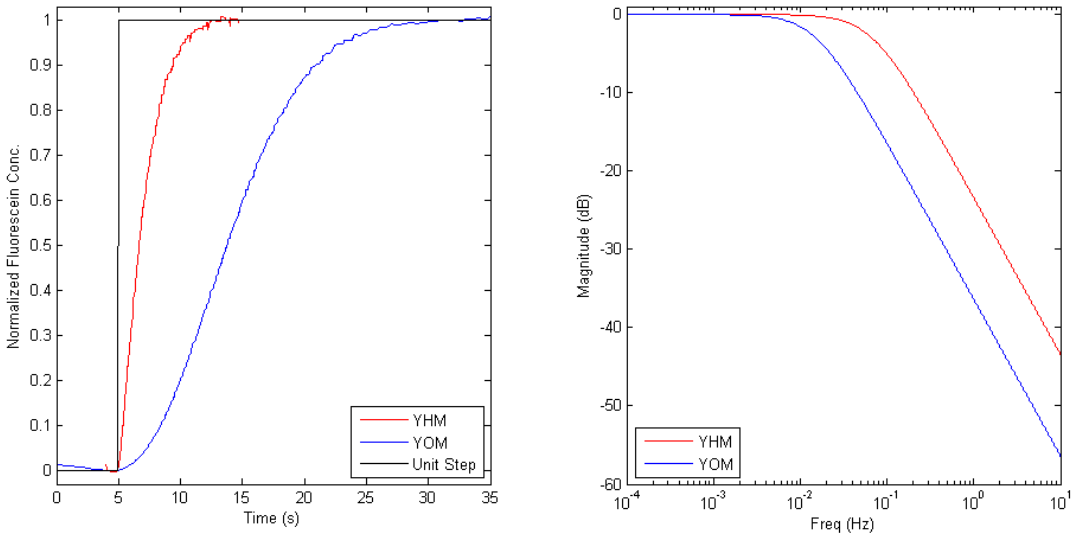

Figure S7. Time and frequency response of *herringbone mixer* and *obstacle mixer*. The data was extracted from square wave waveforms with a 5 Hz PWM signal at frequencies of 50 mHz for *herringbone mixer* (YHM) and 10 mHz for the *obstacle mixer* (YOM)

Concentration waveform generation

After careful characterization and optimization of the individual components necessary for generating concentration waveforms, we assembled the microfluidic system and studied how switching frequency affected the shape of generated waveforms. As the frequency increases, the sawtooth waveform starts to morph into a triangle wave (**Figure S8**) while the square waveform exhibits sharper and more frequent peaks (**Figure S9**). Therefore, it is possible to tune the waveforms by simply controlling the frequency without the necessity of changing the physical system components.

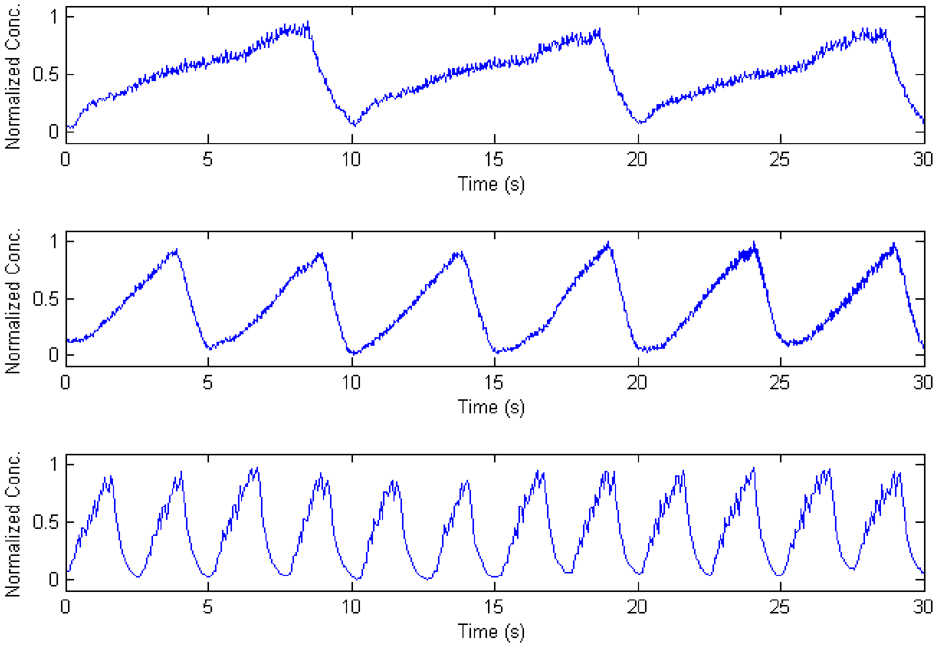

Figure S8. Comparison of 100 mHz, 200 mHz, and 400 mHz sawtooth waveforms from RC2

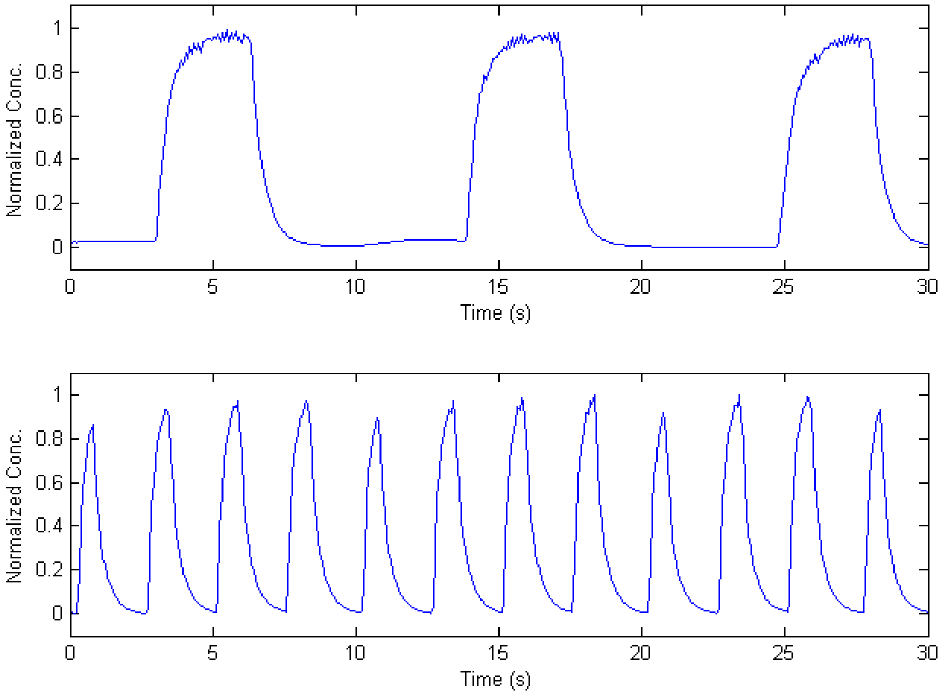

Figure S9. Comparison of 100 mHz and 400 mHz square waveforms from RC2

Data analysis for time and frequency response

The time and frequency responses were analyzed for the *filter chip* and *resistor chip*. For determining the *time-constant* and *cut-off frequency*, a short time-lapse video of the chip's step response was recorded under a fluorescence microscope. Then the video sample was uploaded to ImageJ and the corresponding brightness was extracted into gray values with time stamps.

MATLAB algorithms were then used to analyze the data to manipulate a full waveform into equivalent sections and average them to extract step and frequency responses. Subsequent analysis was completed on the short output waveforms obtained from this script instead of the full waveforms. This script finds the first period of a waveform and uses it as a template in cross-correlation with the entire waveform. The highest values obtained from cross-correlation are the best-matched sections of the waveform to the template. Each section is then averaged together to find the step and frequency response. The MATLAB code is shown below.

```
close all
clearvars -except X Y   %X and Y from data import

t=X';   %Normalizing Data
yP2P = max(Y) - min(Y);
Ynorm = Y - (max(Y) + min(Y))/2;
Ynorm = Ynorm./yP2P;
wave=Ynorm';
% wave = medfilt1(wave, 15);

figure(1)   % finding templates, using first
plot(t,wave,'b')
axis([0,X(end),-0.85,1.15])
hold on;
posN = wave>=0;
plot(t,posN,'r')
bufferBefore = 150;     %%%Change for different waveforms
bufferAfter = 300;      %%%Change for different waveforms
check = 20;             %%%Change for different waveforms
flag = 0;
j=0; k=1;
for i=bufferBefore:length(wave)-bufferAfter
    if flag==0 && posN(i-1)==0 && posN(i)==1
        flag = 1;
        indUp = i;
    elseif flag==1 && j<check
        if posN(i)==1
            j=j+1;
        else
            j=0; flag=0;
        end
    elseif flag==1 && j>=check
        flag = 2; j=0;
    elseif flag==2 && posN(i-1)==1 && posN(i)==0
        flag = 3;
        indDown = i;
```

```
    elseif flag==3 && j<check
        if posN(i)==0
            j=j+1;
        else
            j=0; flag=2;
        end
    elseif flag==3 && j>=check
        flag = 0; j=0;
        ind(k,1:2) = [indUp,indDown];
        k=k+1;
    else
    end
end
template = wave(ind(1,1)-bufferBefore:ind(1,2)+bufferAfter);

figure(2);  % Cross correlation of template to waveform, find peaks
[r,lag] = xcorr(wave,template);
plot(lag,r)
[p,index] = findpeaks(r);
threshold = 80;     %%%Change this for different waveforms
index = index(find(p>threshold))';
p = p(find(p>threshold))';

for i = 1:length(index)
    temp = find(r == max(r(index(i)-200:index(i)+200)));
    if length(temp)>1
        dif = abs(temp - index(i));
        index(i) = temp(find(dif == min(dif)),1);
    else
        index(i) = temp;
    end
end
index = unique(index);
p = r(index)';
iLag = lag(index)';

figure(3)   %plotting template and most matched equivalents
colors = ['b','r','g','k','y','c','m','b--','r--','g--','k--','y--','c--
','m--'];
for i=1:length(index)
    shortW(i,1:length(template)) = wave(iLag(i):iLag(i)-
1+length(template))';
    plot(shortW(i,:), colors(i));
    hold on
end
hold off
shortWmed = median(shortW(:,:));
shortWavg = mean(shortW(:,:));

figure(4)   %plotting the average and median waveform
plot(shortWavg,'b'); hold on;
plot(shortWmed,'r'); hold off;
legend('avg','med')
```

Flow selection valve and PWM signal generation

The pulse width modulation (PWM) signals for the waveforms of interest were generated by a custom MATLAB algorithm. The PWM signal was imported into the Analog Discovery's waveform generator and directly used to control the flow selection valve through switches. This MATLAB code (see below) can generate sinusoidal, square, and sawtooth waveforms but can easily be adapted for any waveform.

```
F2 = 0.1;                      % Freq of waveform
F1 = 5;                        % Freq of PWM
T = 1/F2;                      % Total time = 1 Period
dt = 1/F1/10;
t = 0:dt:T;
A = 5;                         % Amplitude of Sawtooth (arbitrary)
c = A.*sawtooth(2*pi*F1*t);    % Carrier sawtooth

%%% Desired Signals
%   m can be any waveform you want but make sure T above is 1 period long
%   uncomment desired one or create a new one

m = 0.95.*A.*sin(2*pi*F2*t);           % Sine Wave
% m = 1.01.*A.*square(2*pi*F2*t,50);  % Square Wave with duty cycle 0 to
100
% m = A.*sawtooth(2*pi*F2*t,0.5);     % Sawtooth with maximum at loc 0 to 1
%%%

n = length(c);                 % Length of carrier sawtooth is stored to 'n'
pwm = zeros(1,n);
for i=1:n                      % Comparing Message and Sawtooth amplitudes
    if (m(i)>=c(i))
        pwm(i)=1;
    else
        pwm(i)=0;
    end
end
pwmN = zeros(1,n);
pwmN(find(pwm==0))=1;
```
